# Supplementary material for: MetaRibo-Seq measures translation in microbiomes
Source: Nat Commun. 2020 Jun 29;11:3268. doi: 10.1038/s41467-020-17081-z (PMC7324362; doi:10.1038/s41467-020-17081-z)
Supplement: Supplementary file 10 — Supplementary Data 7 [file 41467_2020_17081_MOESM10_ESM.zip › File2/Confidence_High_Taxonomy/290635_out.krona.html]

Javascript must be enabled to view this page.

members
magnitude
magnitudeUnassigned
count
unassigned
taxon
rank

290635\_out

44

2
superkingdom
44

phylum
44
1239

44
class
186801

44
order
186802

186803
family
44

841
44
genus


SRS011084\_contig\_number\_33604SRS011405\_contig\_number\_14487SRS013951\_contig\_number\_14404SRS014235\_contig\_number\_12227SRS014412\_contig\_number\_3086SRS014459\_contig\_number\_31908SRS015065\_contig\_number\_25529SRS015264\_contig\_number\_9283SRS015431\_contig\_number\_56021SRS015890\_contig\_number\_11705SRS016095\_contig\_number\_14558SRS016335\_contig\_number\_3420SRS016753\_contig\_number\_contig-100\_1744.62138SRS016954\_contig\_number\_20775SRS018888\_contig\_number\_423SRS019068\_contig\_number\_18281SRS019161\_contig\_number\_19596SRS019381\_contig\_number\_13123SRS019445\_contig\_number\_1600SRS023346\_contig\_number\_7351SRS024331\_contig\_number\_35971SRS045645\_contig\_number\_25926SRS048164\_contig\_number\_27502SRS048870\_contig\_number\_12411SRS051031\_contig\_number\_12114SRS051031\_contig\_number\_34903SRS058770\_contig\_number\_25037SRS064645\_contig\_number\_11SRS075021\_contig\_number\_6631SRS077454\_contig\_number\_14546SRS098881\_contig\_number\_12087SRS100021\_contig\_number\_2375SRS104693\_contig\_number\_9071SRS105153\_contig\_number\_34532SRS143417\_contig\_number\_537SRS144135\_contig\_number\_32453SRS144506\_contig\_number\_5760SRS146764\_contig\_number\_31912SRS146812\_contig\_number\_contig-100\_1052.322444SRS147766\_contig\_number\_contig-100\_394.122539SRS147977\_contig\_number\_8292SRS148091\_contig\_number\_12965SRS148424\_contig\_number\_25531SRS893327\_contig\_number\_560
360807
44
species
